# Cystoscopy

**Published:** 1911-09-23

**Authors:** Ivor Back

**Affiliations:** Assistant-Surgeon St. George's Hospital, and Surgeon to Out-patients, Royal Waterloo Hospital for Children and Women.


### Hospital Clinics.

### CYSTOSCOPY.

By IVOR BACK, M.A., M.B., B.C., F.R.C.S., Assistant-Surgeon St. George's Hospital, and Surgeon to Out-patients, Royal Waterloo Hospital for Children and Women.

(Clinical lecture delivered at St. George's Hospital, on Tuesday, May 23, 1911.)

Gentlemen,—It is no exaggeration to say that the introduction of the cystoscope has revolutionised urinary surgery. The diagnosis of urinary diseases is now established upon a satisfactory and scientific basis. Previously we had to rely upon careful examinations of the constituents of the urine as the principal means of diagnosing in what part of the urinary apparatus the primary focus of the disease was situated. All that we were able to tell by this means was that we were probably dealing with disease of the bladder or of the kidney: and even that we could not say with anything approaching certainty.

### The Old Methods.

Nor, in the case of the kidney, could we tell which one was abnormal. We were accustomed to rely upon the presence or absence of a swelling or tenderness, palpable or visible through the abdominal parietes, in one or other subcostal region. Quite commonly, both these clinical signs were absent: and yet when the patient died, as frequently was the case, one or both kidneys were found, at the post-mortem, to be in an advanced stage of disease. My own experience is that it is only in quite a small percentage of cases that you are likely to find a swelling in one or other loin. Of course, I am aware that a pyonephrosis or a sarcoma of the kidney in a young child may assume enormous proportions before it eventually proves fatal; but this is the exception rather than the rule. Before cystoscopy was introduced we were faced with another difficulty. In those cases in which we had been successful in diagnosing disease of one kidney, we were not really able to estimate the adequacy of the other (a point of tremendous importance supposing we were about to perform the operation of nephrectomy), because the methods at our disposal were both clumsy and inaccurate; so inaccurate that in the early days of this operation some surgeons used to open the abdomen anteriorly by a transperitoneal incision in order to palpate the opposite kidney before anything further was done; or, alternatively, the opposite kidney was explored through a separate lumbar incision.

### Some of their Fallacies.

I need hardly point out to you the fallacies which might arise from this method. In the first place, a kidney may be functionally inadequate and yet feel perfectly normal; and, in the second, a kidney whose size and contour is unusual may be a completely efficient organ. As an example of that a congenitally lobulated kidney, which is functionally a good organ, may be mentioned. In many cases, however, not even this precaution was taken, and the diseased kidney was removed on the hypothesis that the disease had advanced to such an extent as to render the organ entirely functionless, and that, therefore, the excretory needs of the organism were being satisfactorily carried out by the opposite kidney! As you may suppose, one or two disasters of a drastic nature were recorded, and for the one or two that were recorded it is more than likely that there were many that never blossomed into print. You will find that surgeons are so constituted in every country that they are more disposed to publish their triumphs than their accidents. Hence came the suggestion that a diseased kidney should never be removed primarily, but drained only at the first operation, and any urine which it excreted allowed to come through the wound in the loin, so that an examination of a twenty-four-hours specimen of the urine from the bladder could be made, and as this necessarily came from the other kidney its functional adequacy could be tested. If the amount of urea passed by the other kidney was satisfactory, the nephrectomy could be then completed at a second operation. But here again the method was not perfect, since it not only exposed the patient to two operations, but was open to a fallacy, because it could never be made certain that all the urine from the drained kidney escaped from the loin and that none went down the ureter. Further, in certain cases it had to be presumed that a mere shell of kidney substance, which at the first operation appeared to be quite inadequate with regard to any excretory function, was really doing a considerable amount of work.

### The Luy's Separator.

The next method which came into vogue for estimating the urine from either kidney and so testing its constituents, or the adequacy of the organ which produced it, was known as Luy's separator. This is an instrument shaped like a large English steel bougie. In the concavity of the curve there is a thin rubber diaphragm which can be released by means of a screw in the handle, until it mechanically divides the bladder into two halves. In the shaft there is a channel on either side of this velum, so that the urine expelled from each half of the bladder can, theoretically, be examined separately. This is ingenious in theory but not reliable in practice, since it is practically impossible mechanically to divide the bladder into two compartments so water-tight that urine cannot escape from one to the other. Moreover, it is a cruel instrument, since it is inadvisable to give an anæsthetic; and if no anæsthetic is given the necessary pressure on the trigone to separate the bladder into two halves is such as to cause the patient a considerable amount of pain. In my own experience the instrument has proved most unsatisfactory. In cases of pyuria where, clinically, it was fairly obvious that the pus was coming from one kidney, it has been found in both halves of the bladder after separation. I think I may say, therefore, that this instrument has now been completely discarded in favour of cystoscopy with catheterisation of the ureters.

I have brought here to-day to show you an ordinary irrigating cystoscope and a catheter cystoscope for catheterising the ureters. This irrigating cystoscope which I show you is, I think, the best at present on the market. I purchased this one from Messrs. Meyrowitz, of Bond Street. It consists, as you see, of two parts—a hollow outer tube, which carries at its end the lamp, and an inner tube, which fits it closely and carries the mirror. The outer hollow tube has, near the eye-piece, a little valve which can be easily forced back by the insertion of this small hollow plug, so that the instrument now becomes a metal catheter. When the cystoscope is introduced the inner portion can be removed, the hollow plug inserted, and the bladder thoroughly washed out without any further instrumentation. This is a great advance upon the old method, in which we used first of all to wash out the bladder through an ordinary catheter, and then pass a cystoscope. What frequently happened was that in the subsequent passage of the cystoscope hæmorrhage was set up, which obscured the field of vision.

### Points in Technique.

The technique of cystoscopy is simplicity itself. An anæsthetic is not only unnecessary, but a positive disadvantage, because under an anæsthetic you cannot estimate the distension of the bladder so exactly as the patient can do it for you. I place the patient on his back, with the sacrum raised, either upon a sandbag or, which I find more satisfactory, upon an inverted tin bowl, and for this I use one of those bowls which we have in the theatre for biniodide lotion. This is much preferable to the lithotomy position, because when the patient is doubled up the orifices of the ureters sink backwards into the pelvis, and are much more difficult to find. The glans penis is rendered aseptic, and one or two drachms of a 2 per cent. solution of eucaine lactate are then introduced into the urethra with an ordinary stylographic pen-filler. The external meatus is closed with the finger and thumb of the left hand, while the eucaine is massaged down the urethra as far back as the compressor urethræ. It is necessary to wait three or four minutes before the eucaine has rendered the urethra anæsthetic, and this interval of time may be profitably employed in putting the apparatus together, connecting it to the battery, and adjusting it so that the proper amount of current is going through the lamp. This can be estimated by the fact that the wire filaments of the lamp are only just visible at their bases. The cystoscope is then introduced, the beak of the instrument, whose position is always indicated by the knob on the eye-piece, being kept along the roof of the urethra. When the point of the cystoscope engages under the triangular ligament its end should be lowered gradually, and as this is being done it will be felt to pass into the bladder. Force is not only unnecessary, but positively harmful, since it may tear the prostatic urethra and so set up an amount of hæmorrhage which will render an adequate examination impossible. Whenever difficulty is met with in introducing the cystoscope it is due, in my experience, to the fact that the eye-piece has not been lowered sufficiently. The cystoscope is now converted into a metal catheter in the manner I have shown you, and through it the bladder is thoroughly washed out, until the fluid returns perfectly clear. The bladder is next distended with fluid until the patient expresses a desire to micturate. A normal bladder will hold 10 ounces quite comfortably, but in disease, such as cystitis and tuberculosis, you will find that the amount tolerated is considerably less, sometimes not more than 3 or 4 ounces. The inner tube is then reintroduced, the cystoscope connected to the terminals of the battery, and the light turned on.

The bladder should be examined by a routine method. If this is not done there is a great chance of some pathological condition being overlooked. In the first place the instrument should be passed as far as possible into the bladder with the beak uppermost, and withdrawn gradually until it is near the internal urinary meatus. In this way the anterior wall of the bladder can be seen, though you will usually find that the field is somewhat obscured by bubbles of air which float upon the surface of the fluid in the bladder. In spite of this, however, any gross abnormality would easily be discovered. The orifices of the ureters should next be looked for. The right ureter will be found by turning the cystoscope to such an angle that if the eye-piece is compared with the face of the clock the knob on it points to the figure 8. To find the left ureter the knob must be turned round to the position of four o'clock. In a normal bladder they are seen to be small round apertures. Their appearance is modified by disease. In cases of pyelitis they will be found to be large, and generally to stand out upon the surface of the bladder like small mammillæ, whereas in tuberculous disease of the kidney, for some reason which has not been satisfactorily explained, they are retracted towards the abdominal cavity, so that the opening of the ureter is pulled out like a bell tent. The next thing to observe is the efflux from the ureters. Normally this occurs every fifteen to twenty seconds. As the urine is forcibly discharged from the ureter by muscular contraction it causes a small commotion in the bladder in the neighbourhood of its orifice, and this gives the appearance of a slightly turbid vortex. If no efflux is observed from one or other ureter it is presumable that the kidney on that side is not acting properly. For instance, the ureter may be blocked by a ureteric calculus. In a case of pyonephrosis the ureter on the corresponding side will discharge pus instead of clear urine, and this can be seen as it is squirted into the bladder to resemble a puff of smoke from the funnel of an engine. In cases of malignant disease of the kidney or of renal calculus, the efflux will be red on account of the contained blood, and it will be discharged from the ureter like a red ribbon, which subsequently becomes diffused into the fluid in the bladder. Thus one is able to estimate absolutely, in the case of pus or blood in the bladder, whether the disease is originally vesical or renal, and in the latter case from which kidney it is coming. But here I must give you a word of warning. Hæmaturia is nearly always intermittent, and if on the first occasion when you cystoscope a patient you cannot find blood coming from one or other ureter, you must wait until he is having an attack of hæmaturia and cystoscope him during that attack. Only then can you certainly diagnose disease of one kidney when you have seen blood coming from that ureter. A very interesting case was under my care in this hospital during the last year which illustrates this point.

### An Illustrative Case.

A man, æt. 41, previously healthy, consulted me because he had on two occasions passed blood in his urine. The onset was sudden and absolutely painless. He had no other symptom whatever. Nor could he attribute any cause for the bleeding. His general health was not impaired. There was no abnormal swelling to be felt in either loin. I took him in and cystoscoped him. At that time he was not bleeding. All that I could see was this: the right ureteric orifice was rather small and rather pale. The left was larger and surrounded by a pinkish area. It was therefore presumable that the right kidney was not doing as much work as it should. I sent him out with instructions that the moment he noticed any further hæmaturia he was to come back and be cystoscoped again at once. He returned in about three months at midnight. I took him immediately to the theatre and cystoscoped him, and on this occasion, on looking at the right ureteric orifice I found at each efflux a bright red streak extruded into the bladder. A skiagram was taken and no evidence of renal calculus was obtained. The kidney was not enlarged. I therefore diagnosed that he was suffering from hypernephroma of the right kidney. A catheter was passed up the left ureter, and the adequacy of the left kidney demonstrated. I exposed the right kidney and found the hypernephroma in its pelvis and removed it. There is one small point about which I must warn you. If the patient does not lie absolutely flat the bladder will be stretched over the ureter on his dependent side, and it will form a ridge in it which you may mistake for an abnormality. It is due to nothing more than his uneven position.

### The Bladder Walls.

The lateral walls of the bladder should then be examined in turn by revolving the knob of the eye-piece respectively to three and nine o'clock. A pedunculated papilloma of the bladder, if you see one, is quite an attractive object. It hangs from the wall of the bladder, and its slender processes float in the fluid exactly like a sea anemone seen under water. If it is sessile and of any size it may be extremely difficult to diagnose from an epithelioma. In fact, I do not think that any living surgeon has sufficient experience to say, as the result of cystoscopy, that a given sessile tumour is innocent or malignant. The microscope alone can enable us to give a definite opinion on this question. All you can say is that you have a sessile tumour and that it is or is not operable on account of its size. A malignant growth, however, is generally associated with a certain amount of œdema, and for this reason it may sometimes be difficult to diagnose from a localised patch of cystitis in which œdema is also generally present. To be able to recognise generalised cystitis is a matter of experience. One must have looked at a large number of normal bladders before one can say that a given bladder is inflamed. The ways by which one recognises it, however, are these: In cystitis the mucous membrane of the bladder is pinker than a normal mucous membrane, and in addition the vessels which one sees coursing over it are larger, more congested, and redder than usual. Tubercle and bilharzia will be recognised by the fact that they cause multiple localised raised ulcers. The differential diagnosis can then be surely established by a bacteriological examination of the urine. A vesical calculus is easily recognised as an irregular rounded mass lying in the base of the bladder.

### The Prostate.

Lastly, the condition of the prostate should be examined. If one gradually withdraws the cystoscope, keeping one's eye to the eye-piece and the beak towards the anterior abdominal wall, one will see, as the mirror enters the prostatic urethra, a definitely red projection on either side with, in a normal prostate, a wide angle between the two. If the prostate is enlarged, this wide angle is reduced by the approximation of the two halves to a slit which is not in the middle line, but is pressed to one or other side, according as the enlargement is mainly in the right or left lobe. For instance, if the right lobe is enlarged, the slit will point to the left, and *vice versa.* In some cases, instead of a single slit you will see a V-shaped opening. This is observed in an enlargement of the middle lobe of the prostate.

I now show you a catheter cystoscope. In principle it is essentially the same as the ordinary cystoscope, but it has this difference: that it has a separate canal to pass a catheter down it. The point of the catheter comes out just proximal to the mirror, and its position can be adjusted by means of this knob near the eye-piece. When the ureteric orifice has been located, the position of the apex of the catheter is adjusted until it is opposite the orifice, and then with firm, steady pressure it is pushed on until it enters the ureter, and it can be passed right up into the pelvis of the kidney. If it is desired to estimate the adequacy of the kidney, the cystoscope can be pulled out over the catheter, which is left *in situ*, and the urine which is passed through the catheter is collected in a vessel and a twenty-hour hours' specimen can thus be estimated. It is particularly useful also in corroborating the diagnosis of a stone in the ureter. For this purpose we use a ureteric bougie which is impregnated with oxide of iron, and thus gives a shadow with the $x$-rays. It is passed up the ureter as far as it will go, that is to say until it comes against the impacted calculus, and the patient is then $x$-rayed in the antero-posterior and in the lateral planes, with the bougie in position. If the point of the bougie corresponds to the shadow which the stone gives a diagnosis of a calculus in the ureter may be made with certainty.

